# Cytotoxic T Lymphocytes and Vaccine Development 2013

**DOI:** 10.1155/2013/865314

**Published:** 2013-02-04

**Authors:** Zhengguo Xiao, Kim Klonowski, Hanchun Yang, Julie Curtsinger

**Affiliations:** ^1^Department of Animal and Avian Sciences, University of Maryland, College Park, MD 20742, USA; ^2^Department of Cellular Biology, University of Georgia, Athens, GA 30602, USA; ^3^Department of Veterinary Microbiology and Immunology, College of Veterinary Medicine, China Agricultural University, Beijing 100083, China; ^4^Center for Immunology, University of Minnesota, Minneapolis, MN 55455, USA

CTLs play a critical role in fighting chronic virus infection and cancers. Despite the tremendous efforts spent so far, generating potent and highly effective CTLs remain a major roadblock for disease control (prevention and therapies). Activation of CTLs requires different stimuli and is influenced by different disease conditions. In this special issue, several papers investigated functions of cytokines, such as IL-15 and IL-6, in activation of CTLs. Several papers are focusing on the therapeutic effects of CTLs in different cancers using novel approaches targeting tumor-specific CTLs. In addition, potential markers and mechanisms for rapid progression of AIDS are suggested using SIV model.

 In the first paper entitled “*IL-6 production by dendritic cells is dispensable for CD*8^+^ *memory T-cell generation,*” Daudelin et al. describe a new observation that IL-6 is not required in APC vaccination.

In the second paper entitled “*What is recent in pancreatic cancer immunotherapy?*” Niccolai et al. summarize the current immunotherapy of pancreatic cancer targeting cancer-associated antigens.

In the third paper entitled “*MUC1-specific cytotoxic T lymphocytes in cancer therapy: induction and challenge,*” Roulois et al. review current knowledge regarding MUC1 as a potential target tumor therapeutic vaccines.

 In the fourth paper entitled “*Characterization of CD*8^+^ *T cell responses in the peripheral blood and skin injection sites of melanoma patients treated with mRNA electroporated autologous dendritic cells (TriMixDC-MEL),*” Benteyn et al. report that functional TAA-specific CD8^+^ T cells are detected in both the skin and the peripheral blood after TriMixDC-MEL therapy.

In the fifth paper entitled “*CpG and interleukin-15 synergize to enhance IFN-production by activated CD*8^+^ *T cells,*” Cobb et al. present evidence that IL-15 synergizes with CpG in the induction of IFN-*γ* in activated CD8^+^ T cells, expanding our understanding about the function of IL-15. 

In the sixth paper entitled “*Increased Toll-like receptor signaling pathways characterize CD*8^+^ *cells in rapidly progressive SIV infection,*” Marcondes et al. suggest that TLR overexpression may indicate ineffective T-cell response in rapid progression in HIV infection.

We thank all of the authors for their great contributions to this special issue and appreciate their patience in processing their manuscripts. We also want to thank all of the reviewers who went through the manuscripts multiple times, providing insightful suggestions. We really hope this special issue will continue to thrive as a timely communication platform for basic and translational research on CTLs.

*Zhengguo Xiao* *Kim Klonowski* *Hanchun Yang* *Julie Curtsinger*

